# Heat and law enforcement

**DOI:** 10.1093/pnasnexus/pgad425

**Published:** 2024-05-14

**Authors:** A Patrick Behrer, Valentin Bolotnyy

**Affiliations:** Center for Food Security and the Environment, Stanford University, Stanford, CA, 94305, USA; The World Bank, Development Economics Research Group, Washington, DC, 20433, USA; Hoover Institution, Stanford University, Stanford, CA, 94305, USA

## Abstract

Using administrative criminal records from Texas, we show how high temperatures affect the decision-making of police officers, prosecutors, and judges. We find that police reduce the number of arrests made per reported crime on the hottest days and that arrests made on these days are more likely to be dismissed in court. For prosecutors, high temperature on the day they announce criminal charges does not appear to affect the nature and severity of the charges. Judges, however, dismiss fewer cases, issue longer prison sentences, and levy higher fines when ruling on hot days. Our results suggest that the psychological and cognitive consequences of exposure to high temperatures have meaningful consequences for criminal defendants as they interact with the criminal justice system.

**Significance Statement**

Comprehensive data on arrests and criminal prosecutions in Texas over almost a decade show that heat has pervasive impacts on law enforcement. On hot days, police make fewer arrests relative to the number of crimes and are more likely to make arrests that do not result in successful prosecutions. Judges who rule on cases on hot days are less likely to dismiss cases and more likely to issue longer sentences and higher fines. Overall, heat appears to have adverse impacts on the performance of various actors in the judicial system.

## Introduction

High temperature increases criminal activity (1–9). But what effects does it have on other actors in the judicial process? One explanation for the impact of heat on crime, with broad support in both the psychological and economics literatures, is that heat has cognitive and psychological effects that reduce emotional control and increase aggression (4, 5, 10, 11). An implication of the cognitive and psychological channel, however, is that heat not only impacts potential civilian defendants but also the police charged with arresting them, the prosecutors responsible for prosecuting them, and the judges who ultimately preside over their trials.

In this paper, we examine heat’s impacts in the criminal justice system by focusing on nondefendant actors (i.e. the police, prosecutors, and judges). Heat’s effects on these actors have important implications for how crimes are pursued and for the outcomes defendants ultimately experience. Despite a robust literature on heat and crime, much less attention has been given to how heat impacts the range of nondefendant actors in the judicial system. Some recent work has attempted to address this gap, with varied results. Police appear to reduce effort in the execution of their duties not related to criminal justice (i.e. traffic stops) on hotter days (2, 12), but do not commit more fatal shootings on those days (13). Judges may grant fewer asylum requests on hot days (14), though an examination of additional data on asylum requests has called this result into question (15). Judges in India have been found to issue more convictions on hotter days (16).

Separate work has demonstrated how heat warps decision-making—by increasing people’s irritability, anger, and hostility (4, 5, 17, 18). The argument is succinctly summarized: “aggression in heat is mediated by emotions, cognitions[sic], and stress from affective-thermoregulatory conflict that produces violently aggressive behavior” (19). This is consistent with evidence that heat has much larger impacts on violent crimes, or crimes of passion, than on property crimes (1, 2, 9, 20).

Heat not only has negative impacts on psychological control but also on cognitive and noncognitive skills in a range of settings (21). Heat has been shown to reduce student performance in both short term (22–24) and long term (25). Laboratory studies find that performance of both cognitive and noncognitive tasks declines as temperature increases (26–28). Nonpolice government officials appear to be less zealous in the execution of their duties on hotter days (12) and consumers rely more on heuristics for decision-making when subjected to heat stress (29). The cognitive impacts of heat may be particularly important in the context of a judicial system that often requires cognitively demanding decisions from police, prosecutors, and judges.

Capturing the full effect of heat on potential defendants is important from a welfare perspective. Existing work demonstrates that heat imposes substantial welfare costs by increasing criminal activity. But arrests and incarceration also impose welfare costs, particularly on those who are arrested (30–32). Understanding the extent to which the number of arrests changes on hot days because of heat’s impact on police, as opposed to increases in crime, consequently has important implications for how the welfare costs of heat-driven changes in crime are distributed. For example, if arrests on hot days do not keep pace with increases in crime because of declines in police effort, there is likely a substantial welfare cost being shifted onto victims that could be alleviated by increased police effort.

The overall impact of heat on welfare in the criminal justice system also depends on how heat impacts outcomes for defendants after crimes and arrests have occurred. It is well known that judges can be influenced by apparently extraneous factors, such as the loss of a local college sports team around the time of a ruling (33, 34). Prosecutors are also not free from bias in their decisions (35), although no evidence to date has shown how they are affected by heat. Judges and prosecutors may be influenced by heat for the same reason as civilians and police officers or as workers in other settings. Emotional affect, mood, and cognitive function all impact prosecutorial and judicial decision-making. Heat may influence judge and prosecutor decisions through its impacts on both cognitive and noncognitive functions.

Heat’s effects on emotional control and cognition are likely to manifest differently for different actors in the judicial system due to different mediating factors. Police and prosecutors, for example, tend to work in teams, while judges typically make decisions about cases on their own. Police and judges also make decisions under time pressure, either because they must make immediate decisions about arrests or because they must move quickly through large caseloads. Prosecutors, on the other hand, typically make decisions about charges over the course of multiple days. As a result, one might expect to observe the largest impacts of heat on judges—who typically act alone and under time pressure—followed by police, with the smallest effects on prosecutors.

While prosecutors and judges likely conduct most of their business in buildings with at least partial air conditioning, there are still numerous channels through which heat could impact their decision-making. Most directly, heat can reduce the effectiveness of the air conditioning that is in place. Comprehensive data on AC penetration in Texas courtrooms are not available, but, as late as 2021, there were Texas courtrooms that still relied on window units and did not have central air conditioning. While window units clearly have a mitigating impact, the absence of modern heating, ventilation, and air conditioning (HVAC) systems makes older public buildings less protected against heat even if they nominally possess air conditioning. High temperatures make it more difficult to maintain optimal temperature ranges within these buildings.

Aside from the condition of infrastructure in public buildings in which the law is administered, there are other settings and channels through which heat may impact decision-making in the legal system. Existing work has highlighted that both judges and prosecutors, for example, may be exposed to heat before or during their commute and that heat may also influence judge or prosecutor behavior due to exposure during breaks or by preventing them from going outside during a break in order to avoid exposure (14). This exposure may exert a persistent impact on them throughout the day. Additionally, daytime temperatures are correlated with the prior night’s temperatures, which, when high, have been shown to have adverse impacts on sleep and consequently a person’s behavior on the following day (36). Police officers, though often working in air-conditioned vehicles, are susceptible to the effects of heat through these same channels, as well as through more of the work day spent outside. Thus, even though police, prosecutors, and judges, spend large amounts of time working in climate-controlled environments, heat may still play a role in their decision-making. We leave the decomposition of the effects of heat on decision-making across each of these channels to future research.

### Our approach

We use the most comprehensive dataset yet brought to bear on this topic in the United States (for details on our data, see SI-1). Our data cover the universe of more than 10 million arrests across the state of Texas from 2010 to 2017, with comprehensive information on the subsequent prosecution and trials associated with each arrest. Our data are unique in providing detail at the individual defendant level across a large geographic region and in including information about the actions of police, prosecutors, and judges in each case. The richness of our data allows us to better understand how heat affects human behavior in the judicial system.

Our data contain demographic information on the arrested individual, including their home address, race, and date of birth, as well as information on the charge at arrest. Crucially, these data provide dates associated with major decisions: the date of arrest, the date on which the prosecutor files charge(s), and the date on which the judge makes a ruling. On average, in our data, more than 5 months elapse between the date of arrest and the date of a judge’s decision. Combining these data with detailed daily temperature data allows us to measure the causal effect of heat on the share of crimes resulting in an arrest, the probability of conviction or dismissal, and on decisions made by prosecutors and judges.

Specifically, we estimate a series of models that rely on quasirandom variation in day-to-day temperatures to examine how temperature on the day on which decisions are made (or filed) impacts the outcomes of those decisions. While Texas is generally a warm state, we observe substantial variation in day-to-day maximum temperatures both within and across the counties in our sample (Figs. SI-6.1 and SI-6.2). Our main specification uses the now-standard two-way fixed effects model with binned temperature (25, 37).

Our analysis of the impact of heat on police action goes beyond existing examinations and looks at the effects of heat on core police responsibilities—the investigation and arrest of those committing a wide range of crimes, beyond traffic violations. We utilize two measures in this analysis. First, we examine how arrests compare to reported crimes on hotter vs. cooler days in Houston, Texas’s largest city. Second, we consider the outcomes of defendants who are arrested on hotter vs. cooler days. The first measure serves as a proxy of police effort and forcefulness: if heat makes police more forceful or exert more effort, for example, one would expect to see more arrests relative to reported crimes on hotter days. On the other hand, if heat reduces police forcefulness, one would expect to see fewer arrests relative to reported crimes on hotter days. Our second measure captures the effect of heat on the types of arrests that police make. If heat makes police more forceful, they may be more likely to arrest individuals that prosecutors, operating with more remove from the (literally) hot situation, may find difficult to prosecute. As a result, individuals arrested on hot days may be more likely to have their case dismissed.

Prosecutors have a great deal of discretion in the US legal system (38). They can choose to drop charges, not proceed with charges for lack of evidence, or change charges against a defendant. Our data record information about these decisions. Specifically, we observe whether prosecutors choose not to pursue charges, whether they change the initial charges, and if so, in what direction. These charges are recorded in our data as distinct from the charges recorded by the arresting officers. They are also distinct from decisions made by the judge.

We examine two different aspects of prosecutor decisions to test the hypothesis that high temperatures influence their decisions. First, we consider whether prosecutors change the number of cases they choose to drop or release without prosecution on hot days. Second, we examine whether the prosecutor is more likely to add additional charges beyond the arresting charges and, conditional on adding charges, if they add more additional charges on hot days. Our data indicate all of the charges the defendant faced after their arrest. But they also indicate whether the prosecutor specifically added to those charges—distinct from whether or not the prosecutor increased the level of the arrested charge. For example, we see if a prosecutor adds a resisting arrest charge to a defendant who was initially arrested for being drunk and disorderly. In all analyses, we control for the total number of cases that a prosecutor decides on a given day to address concerns that there may be correlation between the temperature on a given day and the number of cases the prosecutor works through. We also control for defendant characteristics—gender, race, ethnicity—and whether the crime is violent or nonviolent.

Turning to judges, our data and setting allow us to test a wider range of hypotheses around the impact of heat on judges than in previous work that examines asylum requests (14) or conviction decisions (16). We use a much longer sample period than previous work in the United States that includes roughly twice as many cases as analyzed in previous work and addresses concerns about sample size (14, 15). Additionally, there is a greater variety of outcomes for defendants in a criminal case as compared to asylum cases, as well as a range of actions the judge can take besides determining guilt or innocence.

We assess whether judges making decisions on hotter days are more or less likely to dismiss a case against a defendant. Judges are often the most important decision-makers in whether a case is dismissed in the US Convictions, on the other hand, depends on the actions of a larger group of people, including the judge, the prosecutor, and the jury. Dismissals may also occur because witnesses or others fail to show up. This suggests some dismissals are outside of the control of the judges. To the extent this is true, it will add noise to our results, but is unlikely to drive those results. One exception is if defense attorneys are less well prepared on hotter days and so are less successful in arguing for dismissals. Given our results on the impact of heat on prosecutors, however, we believe this is unlikely.

Second, we consider the punishments issued by the courts. We have data on the length of the sentence, the length of probation, and the amount of any fines issued. Fines are separate from court costs that defendants are ordered to repay. We do not have information on the types of punishment a particular case is eligible for, so when we analyze punishments we only consider those cases for which the punishment data are not missing. In all analyses, we control for the total number of cases that a judge hears on a given day, to address concerns that there may be correlation between the temperature and the number of cases the judge hears. We also control for defendant characteristics—gender, race, ethnicity—and whether the crime is violent or nonviolent.

## Results

### The impact of heat on the police

**Fig. 1.**
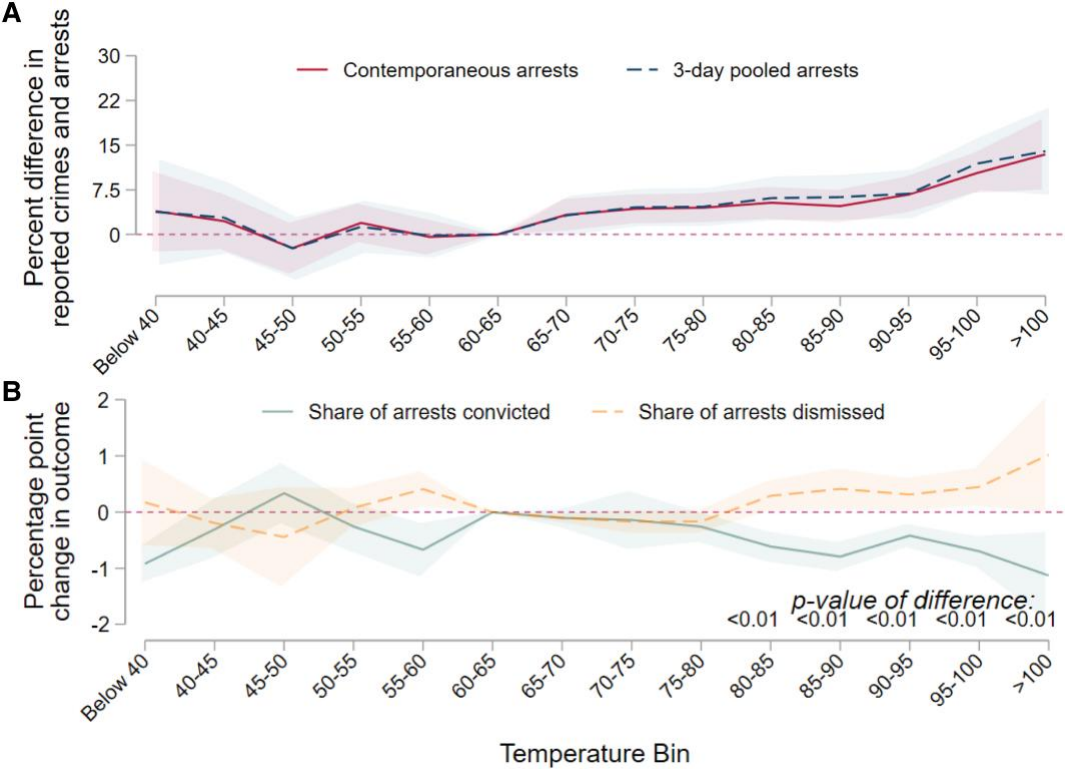
Outcomes related to police behavior on hot days. A) Coefficients from two regressions of heat on the difference between reported crimes and recorded arrests in the Greater Houston area. The solid line considers the difference between reported crimes and arrests on that day. The dashed line considers reported crimes and recorded arrests on the same day plus the subsequent 3 days. In both cases, the difference between reported crimes and arrests grows on hotter days. There are more reported crimes than arrests on a typical day, but on a day with a maximum temperature above $100^{\circ }F$, this difference is roughly 13% larger than on a day with a maximum temperature between 60 and $65^{\circ }F$. Full results of this estimation are reported in Table SI-5.2. B) Coefficients from a regression of heat on the share of arrests that result in a dismissal (dashed line) and conviction (solid line). Temperatures below approximately $80^{\circ }F$ have little effect on these shares. However, a greater share of arrests made on a hot day result in a dismissal relative to arrests occurring on a day with a maximum temperature between 60 and $65^{\circ }F$. Hot days also reduce the share of arrests that result in convictions relative to a day with a maximum temperature between 60 and $65^{\circ }F$. In both panels, the regressions include a full suite of controls for precipitation, county, week, month, and year fixed effects. In both panels, the shaded area indicates the 99% CI.

We start with the effects of heat on our measures of police behavior. We find that arrests respond less to heat than reported crimes. Considering all types of crimes, there are generally three times as many reported crimes as arrests on any given day in our data. To test the impact of heat on police behavior, we examine how the difference between reported crimes and arrests changes on hot days and report the results in Fig. 1A (full results are presented in Table SI-5.2).

We measure the difference between reported crimes and arrests such that a positive difference indicates more reported crimes than arrests. We consider both the number of arrests on the day the crime is reported as well as arrests on the same day the crime is reported plus the subsequent 3 days. In both cases, hot days substantially increase the difference between reported crimes and arrests. On the hottest days, using the contemporaneous results, the difference between reported crimes and arrests is approximately 13% larger than the same difference on cooler days. In Fig. SI-6.3, we show that the delta between reported crimes and arrests is larger on hot days for violent than nonviolent crimes.

We turn now to an examination of how the cases of those arrested on hot days proceed through the judicial system. A significant advantage of our data compared to much of the data used in previous examinations of the impact of heat on crime is that we can observe the outcome of every step of the judicial process—from arrest to prosecution to trial—for a given case. We take advantage of the comprehensive scope of our data to examine whether individuals arrested on hot days experience different outcomes than those arrested on cooler days. In this analysis, we do not consider the temperature on the day of the trial, only the temperature on the day of the arrest.

Arrests increase on hot days but in this analysis, we find that a larger share of these arrests result in dismissals (Fig. 1B). The difference between dismissal and conviction rates begins to appear at temperatures above $80^{\circ }F$ and continues to diverge as temperatures increase. At all temperatures above $80^{\circ }F$, the difference in the change in the share resulting in a dismissal is significantly different from the change in the share resulting in a conviction. We also examine how convictions and dismissals change on hot days for White, Black, and Hispanic defendants. We do not find evidence that the impact varies by race or ethnicity. The change in the relative share of dismissals and convictions is also not the result of different types of crimes occurring on hot days relative to less hot days. Accounting for changes in the types of crime (i.e. violent or nonviolent) that occur on hot and cool days, and corresponding differences in dismissal and conviction rates across violent and nonviolent crimes, leaves 45% of the observed increase in the share of cases dismissed unexplained. We discuss this in detail in Section SI-4. The change in the share of convictions, on the other hand, is almost completely explained by the changing makeup of the types of crimes that occur on hotter days.

We also examine whether the change in conviction or dismissal rates on hot days is different for different crimes. In other words, are violent crimes dismissed at different rates on hot days than violent crimes on colder days? We find no evidence that heat changes dismissal rates or conviction rates differentially for violent and nonviolent crimes. Nor is the impact of hot days on conviction or dismissal rates different for assault, sexual assault, domestic violence, petite larceny, money laundering, or burglary than it is on all crimes. This bolsters the assumption laid out in Section SI-4 that heat does not differentially impact dismissal rates for different types of crimes.

Our findings are likely due to a combination of factors. Reported crime increases are likely driven by actual increases in criminal activity due to heat, as prior work has shown. It is also possible that civilians are more likely to call the police on hot days, either to report actual criminal activity or to report something that is not actually criminal activity. Police, in turn, make more arrests on hot days than on cooler days, but their arrest rate falls further behind the reported crime rate on hot days. This pattern is consistent with a variety of explanations. It could be the case that heat has no effect on police and only effects on crime and/or crime reporting. In this case, one could explain the increasing gap between reported crimes and arrests as the result of capacity constraints on police. As more crimes are reported on hot days, police cannot respond to them all because there are not enough available officers. Alternatively, as suggested by previous work (2), heat may induce police to exhibit less effort on the hottest days, driving the discrepancy between reports and arrests.

Similarly, while our interpretation of the fact that arrests on hot days are more likely to be dismissed is that heat may be having a deleterious effect on police decision-making, there are other possible explanations. As we discuss in SI, different crimes are reported on hot days than on cooler days. It could be the case that police feel more pressure to make arrests to diffuse a situation during the commission of crimes that are more likely on hot days (i.e. violent), even if they know it will eventually result in a dismissal. This kind of behavior would result in the pattern of results we observe, but does not indicate an adverse impact of heat on decision-making. However, such behavior might also lead to differential effects of heat by crime, as police may be more likely to exercise this kind of discretion in the case of alleged violent crimes. We do not observe such differential effects in our data. There may of course also be other, unobserved, features of crimes that occur on hot days that can explain the change in dismissals and convictions in our data.

### The impact of heat on prosecutors

**Fig. 2.**
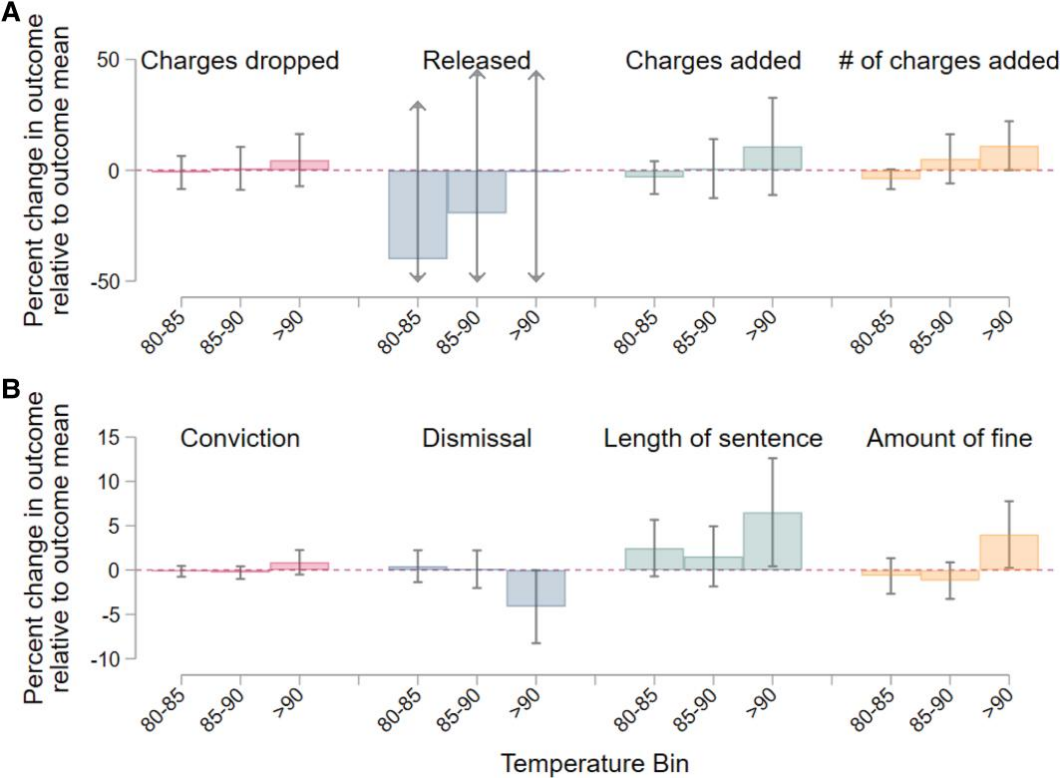
Heat’s impact on prosecutors and judges. A) Coefficients from four separate linear fixed effects regressions of heat on outcomes measuring prosecutor behavior. Outcomes are measured at the case level. We report coefficients from the highest three temperature bins here and outcomes are defined above the coefficient estimates. Standard errors are clustered at the prosecutor level. All include controls for dew point, minimum vapor pressure deficit, and the gender, race, and ethnicity of the defendant. All regressions are weighted by the total cases the prosecutor tries in our sample. “Dropped” refers to cases that are coded in the data as “No Bill,” “Agency drop charge,” “Pros. reject charge,” “Withdrawn by complainant,” and “Pros. rejected charge due to diversion.” “Released” refers to cases that are coded in the data as “Released w/o Pros” and are not coded as “Dropped.” Full regression results are detailed in Table SI-5.3. B) Results from a similar set of regressions but measures outcomes for judges. We include the same set of controls and cluster standard errors at the court level. Conviction indicates the defendant was convicted of the original charge. Dismissal indicates the charge was dismissed. Coefficients indicate the percentage increase in the outcome from the outcome’s mean for an additional day in each bin. In both panels, the gray bars represent the 99% CI. Full results are reported in Table SI-5.4.

We do not find evidence that heat impacts prosecutor decisions regarding whether to drop a case. We show in Fig. 2A (full results in Table SI-5.3) that prosecutors do not appear to release defendants or drop charges with any greater or lesser frequency on hot days. Our point estimates suggest that they may be more likely to add charges on hotter days, but these estimates are very imprecise, with standard errors of the same magnitude as the point estimates. We find that, conditional on adding charges, prosecutors may add more charges on hot days, but our point estimate is only weakly significant and only a small share (roughly 2.5%) of cases in our data ultimately see additional charges being added. If we instead measure heat as the cumulative number of hot days in the 3 days prior to a decision, we find similar results.

When we examine these outcomes separately for White, Black, and Hispanic defendants, we find little to no evidence that heat differentially impacts prosecutors’ treatment of defendants of different races or ethnicities. Our estimates for how heat impacts prosecutors’ decisions to release defendants early, for example, does not vary across race or ethnicity. We do find that prosecutors may be more likely to add charges to Black defendants on hotter days, but our estimates also suggest that conditional on having added charges, White and Hispanic defendants have more additional charges than Black defendants. While meriting future work to examine this question more closely, our results do not suggest that heat leads to differential prosecutorial decisions based on the race or ethnicity of the defendant.

Overall, we find that heat does not exert a meaningful influence on prosecutor decisions. This may be because of the more diffuse decision-making process in most prosecutor offices, making temperature on the day of the decision less relevant for the process. This is consistent with existing work on prosecutor bias, which suggests prosecutors may be biased in specific circumstances (e.g. male prosecutors prosecuting female defendants (35)), but not on average. We do not know which prosecutor in a prosecutor’s office pursued a given case and how the process unfolded, which leaves open the possibility that more refined data might in fact show the impacts of heat on decision-making in specific contexts.

### The impact of heat on judges

Our results indicate that judges consistently behave in ways that are less favorable to defendants when decisions are made on hotter days (Fig. 2B and Table SI-5.4). Our estimate for how convictions change on hot days is imprecise and not significant, but indicates a $90^{\circ }F$ day increases convictions by about 1%. Dismissals, however, fall by just under 5% on a day with mean temperature above $90^{\circ }F$. The fact that convictions are decided through a process involving the prosecutors, jury, and judge, while dismissals tend to be decided by a judge alone, provides further evidence that teamwork can mitigate the effect of heat on decision-making. Although juries also deliberate over numerous days, making our estimate of the effect of heat on their decision-making process imprecise, these findings are in line with the effects of heat on police and prosecutors.

Courts appear to issue more severe punishments on hotter days relative to cooler days. The length of confinement increases by approximately 6.5% when the decision is made on a day with mean temperature above $90^{\circ }F$. Fines also increase on hot days, by approximately 4%, but we do not observe changes in the length of probation.

The number of cases that result in a sentencing decision or a court fine is relatively small. Figure SI-6.4 shows the results of a randomization inference test to examine whether our estimates of the impact of days above $90^{\circ }F$ on sentence length and fines are simply due to random chance in which cases happen to be decided on the hottest days. The *P*-value from the randomization inference test in both cases suggests that our results are significant and not due to random chance in which cases are decided on hot days.

As with prosecutors and police, heat does not appear to impact court decisions differentially based on the defendant’s race or ethnicity. We find that hot days impact decisions about conviction or dismissal similarly for White, Black, and Hispanic defendants. Nor does heat impact the length of sentence or fine amount differently for White, Black, or Hispanic defendants. Our results are also robust to including controls for temperature on the day of the arrest separately from the temperature on the day of the court’s decision. We also examine the impact of heat over the previous 3 days, rather than just on the day of the decision. We find evidence that the effect of heat accumulates if the days leading up to a decision are hot. Finally, we examine the impact of higher previous-night temperatures in addition to day-of-decision temperatures. We find weak evidence that previous-night temperatures may have an impact but, if they do, this impact is small relative to the impact of day-of-decision temperatures.

Taken together, these effects suggest that outdoor temperatures do impact decisions made by judges. Judges issue more severe sentences on hotter days and become less willing to dismiss cases. This is consistent with the hypothesis that heat increases cognitive and emotional stress in ways that have consequences for the outcome of cognitively intensive tasks. Heat can thus have meaningful effects on performance even in settings without physical labor. The effects are lower in magnitude than the effects of heat on judicial decisions in India (16), consistent with the notion that while AC penetration in Texas courtrooms is not complete, it is far greater than in Indian courts. We interpret this difference as representative of the mitigating impact that AC in courtrooms may have on judges. More detailed work examining the role of AC in reducing the effects of heat on cognitively demanding job performance is warranted.

## Discussion

We study how the adverse effects of heat on cognition, mood, and emotional state in turn affect the decision-making process of police officers, prosecutors, and judges. We move beyond existing work on the effect of heat on police by showing that its effects are more complex than just simple reduction in effort. Police make more arrests on hot days, but fewer arrests per reported crime. We thus document the “regulatory gap” caused by heat that has previously been hypothesized (12). We show results that are consistent with a reduction in effort by police on hotter days, as previous work has hypothesized, but our results could also be explained by more binding capacity constraints on the number of officers available to respond on hot days. We also find that arrests made on hot days are more likely to be dismissed relative to arrests made on cooler days. We do not find evidence that this effect varies by whether crimes are violent or nonviolent. We thus provide evidence, consistent with abundant evidence of the negative cognitive impacts of heat (21), that heat may harm the decision-making process of police officers and lead to the unnecessary detention of civilians.

Heat does not appear to impact prosecutorial decision-making. Although judges and prosecutors work in similar environments, prosecutors work on charges over several days and in teams, while judges largely decide on sentence severity alone and often under significant time pressure. That heat appears to impact judges more than prosecutors suggests that teamwork, among other factors, could play an important role in reducing the adverse effects of heat on decision-making. Further research on teamwork and heat would thus be valuable.

There are important limitations to our results. We do not observe police behavior directly, only the consequences of that behavior as it appears in the record of arrests. Our results are consistent with our hypotheses of how and why heat may impact police behavior, but we do not measure direct changes in behavior. There are other explanations that could explain the results we document than the ones we propose. In particular, as noted, police may be subject to more binding capacity constraints on hotter days. It is also possible that the interactions of temperature and unobservable features of crimes are ultimately the ones driving the changes in dismissals and convictions that we document. Finally, police may intentionally make arrests that are unlikely to be successfully prosecuted on hot days to defuse dangerous situations and this behavior could explain the increase in dismissals we observe.

Similarly, we cannot isolate the mechanism through which heat impacts judge behavior. While there are multiple channels through which heat could impact judges—including, but not limited to, exposure during commuting, changed patterns of behavior during the day, and exposure due to imperfect air-conditioning coverage—we do not have direct evidence for these channels. We note, however, that the common perception of judges working exclusively in highly air-conditioned environments does not appear to be true in our setting. While there is no comprehensive database of courthouse air-conditioning penetration in Texas, our review of public information on individual courthouse renovations in Texas indicate that even as late as 2021 courthouses in Texas lacked comprehensive air conditioning.

Our results on prosecutors are also limited because we do not observe the particular race, ethnicity, and gender of prosecutors. Existing work on prosecutor bias has found that while prosecutors may not be biased in general, they can be biased against specific classes of defendants who are unlike them (35). While we do not find evidence that heat impacts prosecutor behavior in general, it remains possible that it exacerbates these types of biases. Evidence from India indicates that the impact of heat on judges varies by gender (16), further suggesting that more detailed examination of prosecutor behavior might uncover evidence of heat’s impacts.

Our results highlight that climate change will have an impact on the criminal justice system apart from its direct impact on the commission of crimes. Taken with the existing evidence of the impact of heat on crime, our results indicate that, absent comprehensive adaptation, a higher frequency of high temperatures could result in worse decision-making by police and harsher decisions made by judges. Our results on police suggest that policy-makers should consider increasing police staffing levels on the hottest days. Whether our results on the discrepancy between reported crimes and arrests on the hottest days are driven by reduced effort or more binding capacity constraints, increasing staffing levels may alleviate the adverse impacts of heat.

Finally, our results lend support to a psychological mechanism for the impact of heat on crime. While other mechanisms may explain the link between heat and the commission of crime, the cognitive and psychological explanation provides a parsimonious theory that unifies both the impacts of heat on the commission of crimes and the impacts we document throughout the judicial system. Heat reduces self-control, negatively impacts mood, increases aggression, and places heightened stress on cognitive faculties. As a consequence, crime increases, police make arrests they likely should not be making, and judges working on tight schedules—as opposed to prosecutors who operate in a team on looser deadlines—make harsher and more punitive judgements. A psychological explanation does not preclude other mechanisms from operating in certain circumstances, including ours, but no other single theory offers a consistent explanation for the full set of these impacts.

## Supplementary Material

pgad425_Supplementary_Data

## Data Availability

The microdata on criminal defendants or court and prosecutor outcomes cannot be made publicly available under our agreement with the Texas Department of Public Safety. To request the raw microdata, contact the department directly (Cassandra Richey—GRP_CJIS_SITE@dps.texas.gov). Code and aggregated data to replicate the tables and figures in the paper will be made available, where possible, on the authors’ websites.

## Preprints

An earlier version of this study was circulated as a working paper titled “Heat, Crime, and Punishment” and was posted on a preprint: http://hdl.handle.net/10986/36881.
