# Supplementary material for: Heat and law enforcement
Source: PNAS Nexus. 2024 May 14;3(5):pgad425. doi: 10.1093/pnasnexus/pgad425 (PMC11093125; doi:10.1093/pnasnexus/pgad425)
Supplement: pgad425_Supplementary_Data [file pgad425_supplementary_data.pdf]

# Heat and Law Enforcement

A. Patrick Behrer[1,2] and Valentin Bolotnyy[3]
[1]Stanford University, Center for Food Security and the Environment, Stanford, California, USA
[2]The World Bank, abehrer@worldbank.org
[3]Stanford University, Hoover Institution, Stanford, California, USA

December 4, 2023

**Abstract**

Using administrative criminal records from Texas, we show how high temperatures affect the decision-making of police officers, prosecutors, and judges. We find that police reduce the number of arrests made per reported crime on the hottest days and that arrests made on these days are more likely to be dismissed in court. For prosecutors, high temperature on the day they announce criminal charges does not appear to affect the nature and severity of the charges. Judges, however, dismiss fewer cases, issue longer prison sentences, and levy higher fines when ruling on hot days. Our results suggest that the psychological and cognitive consequences of exposure to high temperatures have meaningful consequences for criminal defendants as they interact with the criminal justice system.

# Supplementary Information

## SI-1 Materials & Methods

### SI-1.1 Texas Department of Public Safety (TDPS) Data

We start with confidential data from the TDPS that include detailed information about every arrest made in Texas from 2010 through 2017. These data are collected and organized by the TDPS and come directly from specific criminal justice agencies within each Texas county. Arrests are reported by the arresting agencies, prosecutor information is reported by the prosecutors, and the court dispositions are reported by the courts. Data are reported to TDPS every 7 to 30 days, as required by the Texas Code of Criminal Procedures, Chapter 66.252.

Texas state law also requires that counties maintain at least a 90% data completeness rate over a rolling five year period in order to be eligible for certain state funds. Completeness means that the data reflect the most up-to-date status or disposition of each case. We received our data in 2019, so at least 90% of the cases through 2017 have been deemed to accurately reflect their most up-to-date status in our data.[1]

The TDPS arrest disposition data come in several parts. We combine files providing data on the individual arrested, the circumstances of the arrest, details of any prosecution, details of any court trial, and details of the subsequent sentencing or appeal.

The prosecution data can be linked to the individual and arrest data using the unique individual and incident IDs. They include the prosecuting agency, date the prosecutor took action on the case, the action taken, the level of the offense that was prosecuted, and the charge prosecuted. The court data include the court that tried the case, the date of the trial, the final pleading of the defendant, the level of the offense and charge that the court ruled on, the sentence handed down by the court, the length of any court ordered probation or confinement, the amount of any court costs the defendant was ordered to pay, and the amount of any fines the defendant was ordered to pay. The data also include whether the case was appealed and the outcome of the appeal. We link arrest and prosecution charges to the court data using the unique individual and incident IDs.

We drop all arrests and charges for which we do not have court outcome data (i.e., the arrest charge does not have a match in the court data) and charges for which the court has not issued a decision.[1] We also drop misdemeanor C cases as these are inconsistently reported in our data. This leaves us with 2.6 million arrests. We geocode the addresses provided with the address information and match each arrest to the county in which the individual lived when they were arrested. We then collapse the data to the count of arrests at the county-day level. This leaves us with a balanced panel of 742,188 county-day observations from 2010 through 2017.

[1] These are indicated as cases where the result is "pending" or "no determination." Dropping non-matching court cases drops 11% of the arrests in our raw sample.

## SI-1.2 Crime Reports from the Houston Police Department

We supplement our TDPS data on arrests with daily data from the Houston police department, the largest city police department in Texas and the fifth largest by officer count in the United States,[2] on reported crimes. These data report the date, hour, location, and type of crime committed from 2010 through 2018. Importantly, they include reported crimes that do not have an associated arrest and that therefore do not appear in the TPDS data. We geocode the provided locations to match the incidents to the U.S. Census tracts associated with each address. Addresses in the Houston PD data correspond to the location from which each report was filed – not, as in the TDPS data, to the address at which the defendant lived at the time. To account for this, and to account for the fact that defendants may commit crimes in Houston even if they do not live in Houston, we create a sample of arrests from the TDPS data that matches the geographic and temporal coverage of the TPDS incident data. We do so by pulling all arrests between 2010 and 2017 where the address of the defendant was in one of the five counties of the greater Houston area. We match these addresses to census tracts as well, in order to facilitate comparisons between reported incidents and arrests.

## SI-1.3 Weather Data

We match our daily arrest counts with daily weather data from the PRISM Climate Group's gridded re-analysis product. The PRISM product provides daily information on minimum and maximum temperature, minimum and maximum vapor pressure deficit, dew point, and precipitation on a 4km by 4km grid for the continental United States. We aggregate these measures to the county level by taking the average across the grid points within the county. We assign daily maximum temperature to one of 12 5°F temperature bins from 40°F up to 100°F. Days below 40°F and above 100°F are included in separate bins. We also bin daily precipitation to control for the impacts of particularly rainy days. We assign days to four exclusive precipitation bins: no precipitation, less than half an inch, one half to one inch, and more than one inch.

## SI-1.4 Summary Statistics

In Table SI-5.1 we present summary statistics for our primary measure of temperature - daily maximum temperature - for aggregate crimes, and for aggregate crimes by race and ethnicity. Roughly 60% of the days in our sample experience a maximum temperature above 70°F and the majority of days in the sample have no precipitation. We summarize the spatial distribution of hot days in Figure SI-6.1. Arrests are broadly distributed across the state.

High temperature is also evenly distributed across the state. We show the average annual number of days over 90°F. Counties in the Rio Grande Valley have, on average, the largest number of these days, but every county in Texas experiences at least 40 such days in an average year. Figure SI-6.2 underlines the variation in temperatures within counties across years in our sample and across months within a given year. Panel A shows the number of days above 90°F in each year of our sample for three counties selected from each tercile of the distribution of 90°F+ days. While

there is clear separation in the number of days as you move down the distribution - Taylor County never experiences a year with as many hot days as the coolest year in Starr County, and Aransas County experiences only one year matching Taylor's coolest year - there is also clear variation within each county across years in the number of hot days. On average these three counties experience yearly deviations of as many as 25 days on each side of their average number of 90°F+ days.

Looking at the distribution of hot days within the same three counties across months of the year, it is clear there is also variation in when days become hot and cease to be hot within a year. Starr County experiences 50 such days in March during our sample, while Aransas and Taylor experience almost no such days in March. All experience a substantial number of 90°F+ days in August, but while these decline to zero by October in Aransas it takes until January to reach zero days above 90°F in Starr.

# SI-2 Empirical Approach

In all of our analyses, we rely on day-to-day variation in local temperatures within a county to identify the impact of hotter temperatures on our outcomes of interest. Identification rests on the assumption that day-to-day variations in temperature within a county are plausibly exogenous with respect to our outcome of interest. We control for annual trends and month-to-month seasonality in temperature.

## SI-2.1 Analysis of Outcomes in the Justice System

In our analysis of we take the standard empirical approach and estimate a linear fixed effects model with various temperature and precipitation bins. We focus on individual cases and estimate regressions of the form

$$Y_{pidmy} = \beta_k \sum T_{idmyk} + \rho_l \sum R_{idmyl} + \delta_y + \psi_i + \eta_d + \Omega_m \quad (1)$$

where $T_{idmyk}$ is an indicator for whether the mean temperature, in the prosecutor and court analysis, or maximum temperature, in the police analysis, in county $i$ on day $d$ in month $m$ and year $y$ is in the $k^{th}$ temperature bin. We use one bin for temperatures below 40°F and one for those above 90°F. Bins in between are in 5°F increments and we omit the 60-65°F bin. In keeping with,[3] we focus on the mean temperature, rather than the daily max, because mean temperature is more likely to capture high temperatures during the morning commute.

Maximum temperature, in contrast, generally captures the temperature during the peak of the afternoon, when judges and prosecutors are likely to be least exposed to the heat.[2] We use maximum temperature in the police analysis because police are likely to be operating outside throughout the day, including at the hottest parts of the day. In all judge and prosecutor regressions, we also control for the total number of cases that the prosecutor filed or judge heard on that day to account for any

[2]Using max temperature, however, produces qualitatively similar results to using mean temperature.

instances in which having to work through a large wave of cases might influence their behavior. We link prosecutor offices and the courts to counties according to Texas data on where each prosecutor or court is based, in order to assign daily temperatures.

$R_{idmyl}$ is an indicator for whether the day falls in the $l^{th}$ precipitation bin. We omit the highest bin in our estimation. $\eta_d, \Omega_m, \delta_y$, and $\psi_i$ are day-of-week, month, calendar year, and county fixed effects. Our county fixed effects absorb any time invariant location specific determinants of crime. Our daily and monthly fixed effects account for variation in crimes over the course of a week (e.g., there may be more crimes on Fridays) and the year (e.g., there is less outdoor activity in the winter and generally lower crime). Our results are robust to several alternative sets of fixed effects, including a month $\times$ year fixed effect.

$Y_{pidmy}$ represents our outcome of interest for defendant $p$ (e.g., an indicator for whether an arrest resulted in a conviction or the length of defendant $p$'s sentence). Again, our identification rests on plausibly exogenous variation in the temperature on the day of the arrest for defendant $p$ net of any year, month, or day of the week specific variation in temperature or outcomes. In our analysis of prosecutor and judicial decision-making, $T_{idmyk}$ represents the temperature on the day that the prosecutor or judge made a decision in the case of defendant $p$. Our outcome of interest is again $\beta_k$, which in this specification estimates the increase in the probability that a case arrested on a hot day (or decided on a hot day, depending on the analysis) experiences a given judicial outcome $Y_{pidmy}$. In our main specifications of prosecutor and judge outcomes we do not control for temperature on the day of the arrest - relying instead on the fact that temperatures on the day of arrest and temperatures on these decision days are not highly correlated, likely because they occur an average of five months apart. In robustness checks we do control for these temperatures and our results do not change.

When we evaluate prosecutorial and court discretion, we only consider those cases that have reached a particular stage of the judicial process. For example, the share of cases where charges are added by prosecutors are calculated as the number of cases with added charges as a share of the number of cases that prosecutors choose to pursue.

# SI-3 Framework

To clarify the differences between considering reported crimes and arrests, consider the following analytic framework. We express arrests (A) as a function of criminal (C) and police (P) activity, which in turn are determined jointly in equilibrium and depend, in part, on temperature:

$$Arrests = A(C, P) \tag{2}$$

How do arrests evolve with changes in temperature (T), which we define as deviations from the optimum temperature? It will depend on the combined impact of temperature on criminal and police activity.

$$\frac{dA(C,P)}{dT} = \frac{\partial A}{\partial C}\left[\underbrace{\frac{\partial C}{\partial T}}_{(1)} + \underbrace{\frac{\partial C}{\partial P}\frac{dP}{dT}}_{(2)}\right] + \frac{\partial A}{\partial P}\left[\underbrace{\frac{\partial P}{\partial T}}_{(3)} + \underbrace{\frac{\partial P}{\partial C}\frac{dC}{dT}}_{(4)}\right] \tag{3}$$

The four terms on the right hand side capture different aspects of the relationship between heat and arrests. Terms one and two capture the direct impact of heat on criminal activity and the "rational criminal" response to temperature: term 1 captures the direct impact of heat on criminal defendants. Term 2 reflects how crime changes in response to changes in police activity driven by temperature changes. The total effect of these two terms is the object most existing work on heat and crime, using data on reported crimes, has estimated.[3] Term three captures the direct impact of heat on police activity (the effect estimated by ref.[5]). Term four captures any changes in police effort in response to changes in crime due to heat: if, for example, police increase patrols on hot days because they know crime increases on these days.

Heat may impact police activity for many of the same reasons that it impacts criminal activity. Ref.[5] finds police are less active in the heat, arguably because exerting effort on hot days is more costly. This is consistent with a broad literature that finds reductions in labor supply and productivity on hot days in a variety of settings.[6,7] If these negative impacts dominate any change in behavior due to anticipated changes in crime this would manifest as an overall negative sign on term four.[4]

Heat may also, however, make the police more likely to arrest individuals relative to cooler days (i.e. term 3 may be positive). There are at least two reasons for this. If heat increases aggression and violence in the commission of crimes, police may pre-emptively arrest individuals to defuse a situation that heat-driven aggression has exacerbated in a way that would not have occurred on a cooler day. Police officers may also arrest more frequently on hotter days because the officers themselves become more aggressive. Existing work suggests that police are negatively impacted by hot temperatures in ways that make them more aggressive, more tense, and produce more negative views of defendants.[8] Heat also appears to increase out-group bias[9] and may strengthen the pre-existing biases of police officers.

## SI-4 The mechanical effect of crime composition on dismissal rates

What is driving the change in dismissals? One possibility is that different crimes have different rates of dismissal and conviction and heat impacts those crimes differently. Existing work shows that violent crimes increase substantially on hot days while non-violent crimes are less responsive.[10] This implies that the violent crime share of arrests is higher on hot days than on less hot days. If violent crimes are dismissed at higher rates than non-violent crimes, we might see this pattern

[3] The best estimates of term two suggest that it is zero or close to zero and the majority of the existing effect operates through term one.[4]

[4] Ref.[4] use data on instances when LAPD officers leave their cars and find that this actually appears to increase on hotter days, suggesting that term four may be slightly positive. They do confirm a decline in traffic stops, consistent with ref..[5]

simply because of the change in the type of crimes that occur on hot days. Violent crimes are also dismissed at higher rates and convicted at lower rates than non-violent crimes. To what extent does this drive our results?

Our estimates suggest that on days greater than 100°F, the share of arrests for violent crimes as a percent of total arrests increases from 15% to 17%. If we assume that the share of violent crimes that is dismissed remains constant across hotter and cooler days, that implies a mechanical 0.65 percentage point increase in dismissals due to the change in the types of crimes that occur on hot days. We observe an increase in dismissal rates of 1.01 percentage points on hotter days relative to cooler days. So it appears that the mechanical change in dismissals can explain roughly 65% of the increase that we observe. The implied mechanical decline in the convictions rate, on the other hand, is roughly 100% of the observed decline in convictions. The change in convictions is thus due primarily to the changing make-up of crimes on hot days rather than the changing behavior of prosecutors or judges. The implied mechanical changes are based, however, on the assumption that the rate at which violent crimes are convicted or dismissed remains constant across arrests on hot and cold days. Our evidence supports this assumption, but it is difficult to test its validity.

We also examine whether the increase in dismissals is driven by a potential increase in arrests of first-time offenders on hot days and judges or prosecutors exhibiting leniency toward these first-time offenders. We find no evidence that hot days increase the number of first-time offenders or that these cases are driving the increase in dismissals on hot days. We also control for the number of cases a prosecutor issues decisions on and a judge hears on the same day. Doing so, we find no evidence that being arrested on a hotter day means one's case is decided when prosecutors or judges have higher workloads.

# SI-5 Additional Tables

**Table SI-5.1:** Summary statistics

| | Mean | SD | Min | Max |
|---|---|---|---|---|
| **Annual averages of weather measures** | | | | |
| T above 100F | 17.10 | 20.18 | 0 | 138 |
| T 95-100F | 36.75 | 14.41 | 0 | 94 |
| T 90-95F | 49.50 | 13.36 | 8 | 102 |
| T 85-90F | 45.26 | 12.17 | 13 | 121 |
| T 80-85F | 42.94 | 10.34 | 17 | 80 |
| T 75-80F | 37.18 | 9.06 | 13 | 87 |
| T 70-75F | 31.75 | 7.15 | 11 | 60 |
| T 65-70F | 27.26 | 6.24 | 9 | 46 |
| T 55-60F | 17.07 | 5.33 | 2 | 37 |
| T 50-55F | 13.06 | 5.32 | 1 | 31 |
| T 45-50F | 9.15 | 4.48 | 0 | 24 |
| T 40-45F | 6.50 | 3.99 | 0 | 21 |
| T below 40F | 8.89 | 8.15 | 0 | 38 |
| Days with no prec | 232.53 | 31.23 | 125 | 313 |
| Days with less than 0.5 in | 19.67 | 7.49 | 1 | 64 |
| Days with 0.5 to 1 in | 5.78 | 2.70 | 0 | 17 |
| Days with>1in | 107.27 | 28.44 | 25 | 201 |
| **Daily crime averages** | | | | |
| Total crimes | 3.24 | 11.10 | 0 | 213 |
| Violent crimes | 0.57 | 2.10 | 0 | 46 |
| Non-violent crimes | 1.59 | 5.65 | 0 | 137 |

NOTES: We aggregate our weather variables to the annual level and report averages across all counties and years in the sample. Thus, "Mean", for example, indicates the average number of annual days in a temperature bin across all counties and years in the sample. Daily crime average statistics are daily averages across all Texas counties.

**Table SI-5.2:** Impact of heat on the difference in reported crimes and arrests in Houston

| | Contemporaneous arrests | 3-day pooled arrests |
|---|---|---|
| T above 100F | 0.045 | 0.047 |
| | (0.010) | (0.012) |
| T 95-100F | 0.034 | 0.040 |
| | (0.005) | (0.007) |
| T 90-95F | 0.022 | 0.023 |
| | (0.005) | (0.007) |
| T 85-90F | 0.016 | 0.021 |
| | (0.004) | (0.006) |
| T 80-85F | 0.018 | 0.020 |
| | (0.004) | (0.006) |
| T 75-80F | 0.015 | 0.015 |
| | (0.004) | (0.005) |
| N | 1,840,860 | 1,839,600 |
| Outcome mean, T60-65 | 0.33 | 0.03 |
| **Fixed Effects:** | | |
| Tract | Yes | Yes |
| Month | Yes | Yes |
| Year | Yes | Yes |
| DOW | Yes | Yes |

Notes: All columns report the results of a linear fixed effects specification. We estimate the impact of a hot day on the difference between the number of incidents reported to the Houston Police Department (Houston PD) and the number of arrests reported to the Texas Department of Public Safety (TDPS). In all cases we aggregate the count of incidents (Houston PD) data or arrests (TDPS data) to the tract-day level and conduct analysis at that level of aggregation. The sample in all cases is a balanced panel of tracts that contain at least one Houston PD crime report at the daily level from 2010 to 2017. In column 2, we pool arrests across the day of interest and the following two days. Errors are clustered at the tract level and are reported in parentheses. All regressions are weighted by the total population in each tract-year. All regressions include the full set of precipitation bins and temperature bins. Coefficients report the raw change in the difference between incidents and arrests for a day in a given temperature bin relative to the omitted 60-65°F bin. Postive differences indicate more incidents than arrests. 100× the coefficient estimates divided by the mean reported at the bottom of the table indicates the percent change in the difference on days in each bin relative to a day in the omitted 60-65°F bin.

**Table SI-5.3:** Impact of heat on day of prosecution action on filed charges

| | Dropped | Released | Added charge | Number of added charges |
|---|---|---|---|---|
| T above 90F | 1.613 | -0.000 | 0.278 | 0.158 |
| | (2.015) | (0.005) | (0.274) | (0.076) |
| T 85-90F | 0.300 | -0.002 | 0.020 | 0.073 |
| | (1.649) | (0.003) | (0.167) | (0.077) |
| T 80-85F | -0.355 | -0.004 | -0.086 | -0.058 |
| | (1.269) | (0.003) | (0.093) | (0.031) |
| N | 1,992,677 | 1,992,677 | 1,992,677 | 51,321 |
| | | | | |
| Outcome mean: | 35.18 | 0.01 | 2.58 | 1.42 |
| Fixed Effects: | | | | |
| County | Yes | Yes | Yes | Yes |
| Month | Yes | Yes | Yes | Yes |
| Year | Yes | Yes | Yes | Yes |
| DOW | Yes | Yes | Yes | Yes |

NOTES: Standard errors are clustered at the prosecutor level. Outcome for charges is specified in column headings. All regressions are linear probability panel fixed effects. All include controls for dew point, minimum vapor pressure deficit, and the gender, race, and ethnicity of the defendant. All regressions are weighted by the total cases the prosecutor tries in our sample. "Dropped" refers to cases that are coded in the data as "No Bill," "Agency drop charge," "Pros. reject charge," "Withdrawn by complainant," and "Pros. rejcted charge due to diversion." "Released" refers to cases that are coded in the data as "Released w/o Pros" and are not coded as "Dropped."

**Table SI-5.4:** Impact of heat on courts

| | Outcomes | | Punishments | |
|---|---|---|---|---|
| | Conviction | Dismissal | Confinement | Fines |
| T above 90F | 0.609 | -1.216 | 0.065 | 0.040 |
| | (0.464) | (0.588) | (0.030) | (0.018) |
| T 85-90F | -0.195 | 0.030 | 0.016 | -0.012 |
| | (0.242) | (0.304) | (0.016) | (0.010) |
| T 80-85F | -0.096 | 0.128 | 0.025 | -0.007 |
| | (0.204) | (0.258) | (0.015) | (0.010) |
| N | 1,140,602 | 1,140,602 | 763,199 | 1,071,518 |
| | | | | |
| Outcome mean,: | 69.12 | 29.45 | 578.71 | 546.83 |
| Fixed Effects: | | | | |
| County | Yes | Yes | Yes | Yes |
| Month | Yes | Yes | Yes | Yes |
| Year | Yes | Yes | Yes | Yes |
| DOW | Yes | Yes | Yes | Yes |

NOTES: Standard errors are clustered at the court level and shown in parentheses. Outcomes are specified in the column headings. Conviction indicates the defendant was convicted of the original charge. Dismissal indicates the charge was dismissed. In columns 1 and 2, outcomes are measured as the percentage of cases with that result. For example, 29.45% of cases are dismissed. Coefficients indicate the percentage point increase in the outcome for an additional day in each bin. In columns 3 and 4, Confinement and Fines outcomes are logged so that coefficients should be interpreted as percentage changes from the non-logged mean presented in the middle of the table. Confinement is measured in days, fines are measured in dollars. All regressions are linear panel fixed effects. We include the full set of temperature and precipitation bins in all regressions, but suppress some coefficients for readability. All regressions include controls for the total number of cases heard in the day, dew point, and vapor pressure deficit minimum.

## SI-6 Additional Figures

**Figure SI-6.1:** Map of Days with Maximum Temperature > 90°F

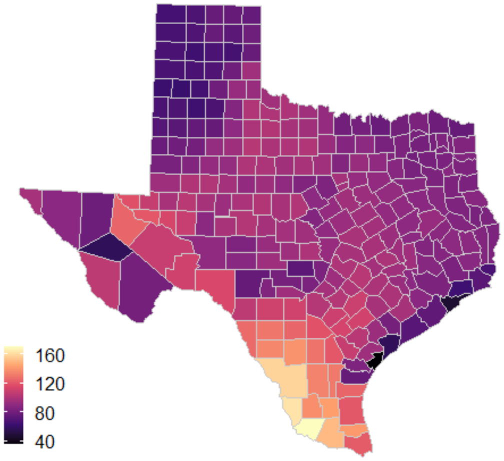


Notes: The average number of annual days with maximum temperature over > 90°F by county over the full sample period.

**Figure SI-6.2:** Hot day distributions

(a) Annual hot days

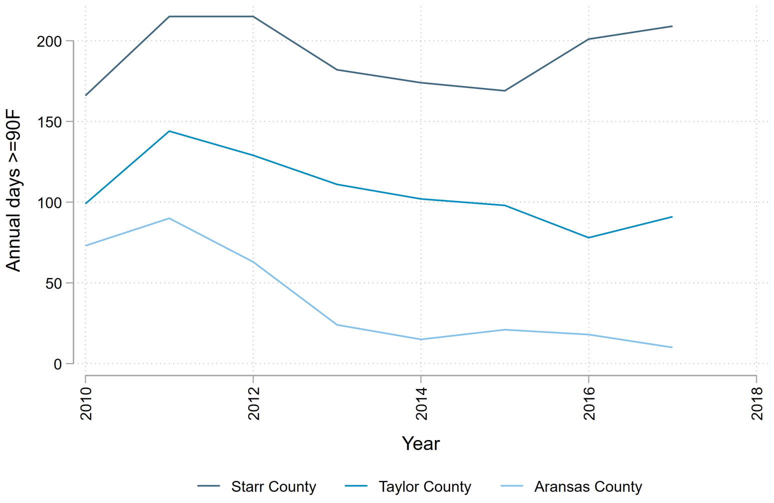


(b) Monthly hot days

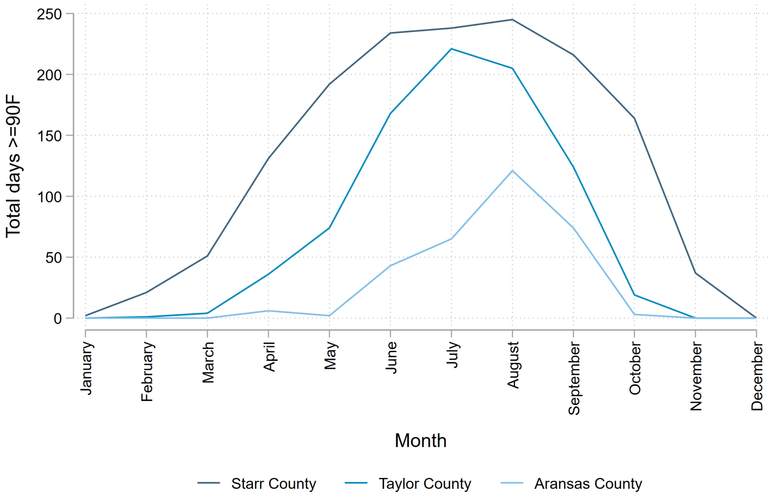


Notes: Panel A shows the trend in days > 90°F in three selected counties from each tercile of the distribution of the average number of hot days over the sample. Panel B shows the trend on average by month for the same counties to illustrate that there is significant variation across counties in our sample – both in the number of hot days from year to year and in the timing of those hot days throughout the year.

**Figure SI-6.3:** Difference in reported crimes and arrests for violent and non-violent crimes

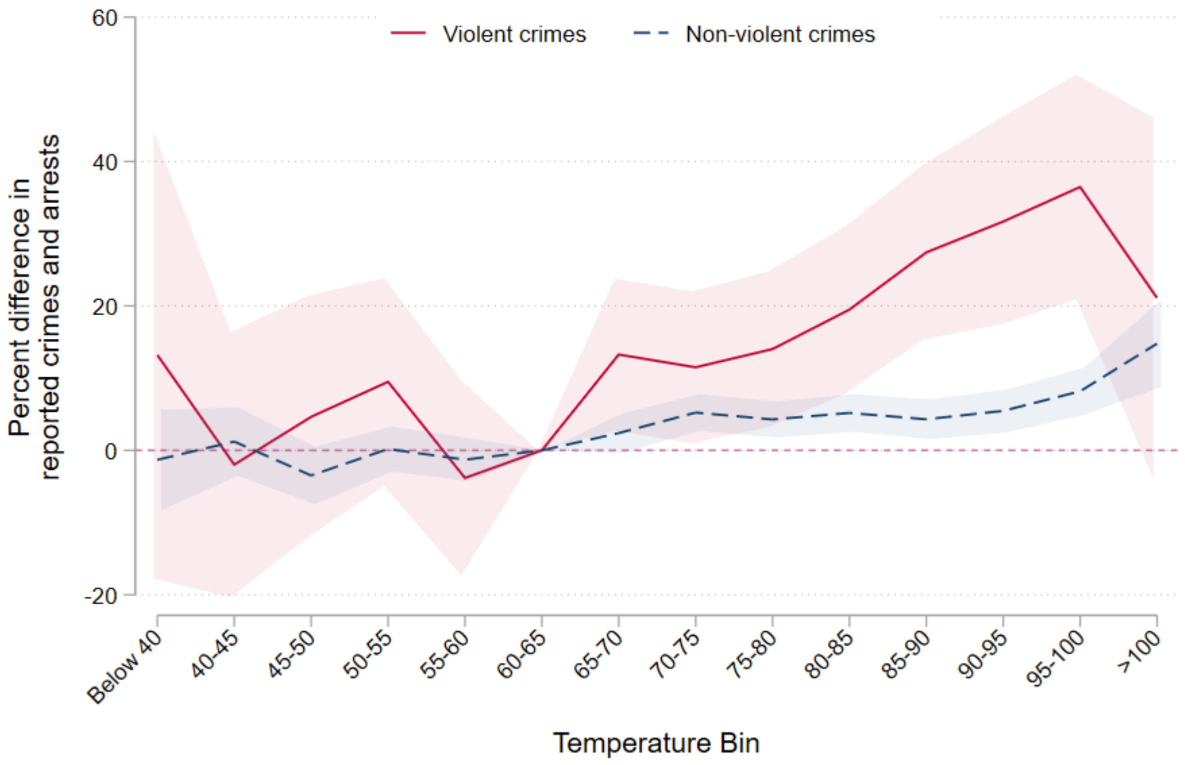


Notes: This figure replicates the 1-day analysis from Figure 1A from the main text, but instead of reporting the effect of heat on the difference between all reported crimes and all arrests, we show the difference separately for violent crimes and non-violent crimes.

**Figure SI-6.4:** Randomization inference tests

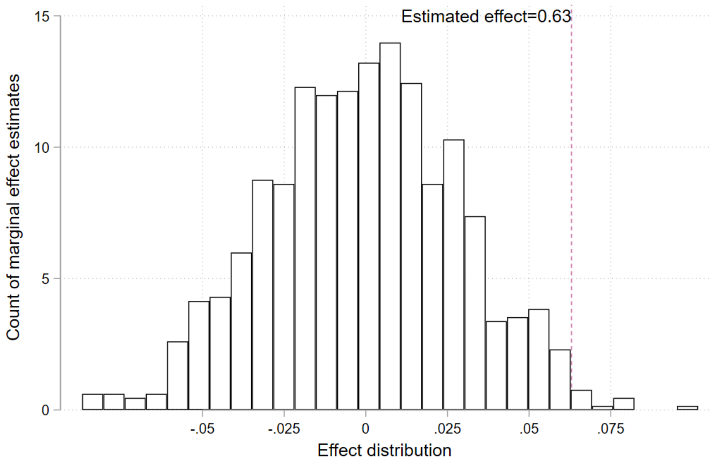


Outcome: length of confinement

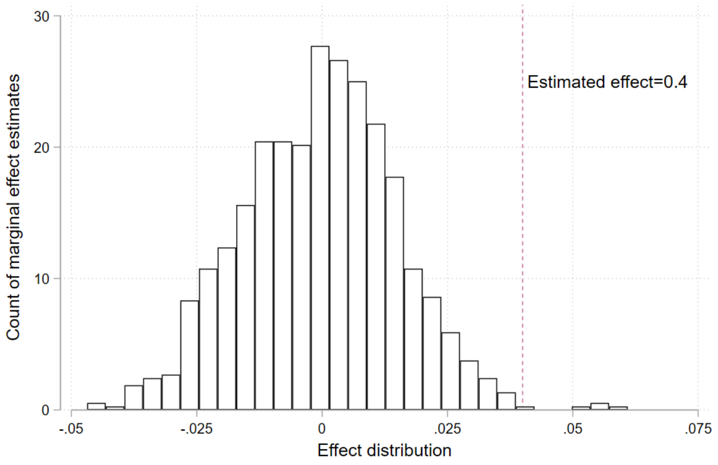


Outcome: amount of court fines

Notes: We re-estimate the impact of heat on the day of a judge's decision on each outcome 1,000 times, re-assigning temperatures randomly across days but preserving the overall distribution of temperature days. This generates a distribution of estimated effects centered on a null effect of zero. We observe that our true estimated effect is well outside this distribution, suggesting that it is not the result of random chance in the cases that happened to be decided on particularly hot days.

# References


[1] Texas Department of Public Safety. *Seventeenth Report Examining Reporting Compliance to the Texas Computerized Criminal History System*. Tech. rep. 2019.

[2] Brian A Reaves and Matthew J Hickman. *Census of state and local law enforcement agencies, 2008*. US Department of Justice, Office of Justice Programs, 1998.

[3] Anthony Heyes and Soodeh Saberian. "Temperature and Decisions: Evidence from 207,000 Court Cases". In: *American Economic Journal: Applied Economics* 11.2 (2019), pp. 238–65.

[4] Kilian Heilmann and Matthew E Kahn. *The Urban Crime and Heat Gradient in High and Low Poverty Areas*. Tech. rep. National Bureau of Economic Research, 2019.

[5] Nick Obradovich, Dustin Tingley, and Iyad Rahwan. "Effects of Environmental Stressors on Daily Governance". In: *Proceedings of the National Academy of Sciences* 115.35 (2018), pp. 8710–8715.

[6] Joshua Graff Zivin and Matthew Neidell. "Temperature and the allocation of time: Implications for climate change". In: *Journal of Labor Economics* 32.1 (2014), pp. 1–26.

[7] E Somanathan et al. *The impact of temperature on productivity and labor supply: Evidence from Indian manufacturing*. Tech. rep. Indian Statistical Institute, New Delhi, India, 2015.

[8] Aldert Vrij, Jaap Van der Steen, and Leendert Koppelaar. "Aggression of police officers as a function of temperature: An experiment with the fire arms training system". In: *Journal of community & applied social psychology* 4.5 (1994), pp. 365–370.

[9] David Blakeslee et al. "In the heat of the moment: economic and non-economic drivers of the weather-crime relationship". In: *Working Paper* (2018).

[10] Matthew Ranson. "Crime, weather, and climate change". In: *Journal of environmental economics and management* 67.3 (2014), pp. 274–302.
